# Mental health trajectories of Brazilian health workers during two waves of the COVID-19 pandemic (2020–2021)

**DOI:** 10.3389/fpsyt.2023.1026429

**Published:** 2023-03-23

**Authors:** Flávia de Lima Osório, Antônio Waldo Zuardi, Isabella Lara Machado Silveira, José Alexandre de Souza Crippa, Jaime Eduardo Cecílio Hallak, Karina Pereira-Lima, Sonia Regina Loureiro

**Affiliations:** ^1^Ribeirão Preto Medical School, University of São Paulo, Ribeirão Preto, SP, Brazil; ^2^National Institute for Science and Technology (INCT-TM, CNPq), Brasília, Brazil; ^3^Department of Psychiatry and Medical Psychology, Federal University of São Paulo (UNIFESP), São Paulo, Brazil

**Keywords:** COVID-19, mental health, workers, anxiety, burnout, follow-up

## Abstract

This study aimed to: (a) monitor the progression of symptoms of mental health burden among frontline workers caring for COVID-19 patients in Brazil during the two waves of the pandemic, considering the number of new cases and deaths, and; (b) to verify the different mental health outcomes and potential associations with current burnout symptoms. A non-probabilistic sample of health professionals was assessed as the pandemic progressed in Brazil (May/2020 August/2021). Standardized instruments focusing on anxiety, depression, insomnia, post-traumatic stress, and burnout symptoms were applied online. The results indicate a decrease in anxiety levels, what was related to when the number of new cases declined (end 1th-wave); symptoms returned to higher levels later. Emotional exhaustion increased when there was a higher incidence of cases, returning to the baseline levels at the end of the second wave. Depersonalization symptoms increased in this phase, characterized by a further decrease in new cases, while professional accomplishment decreased during the follow-up. The highest number of new cases was associated with a higher frequency of anxiety (OR = 1.467;95%CI = 1.109–1.941; *p* = 0.007) and professional accomplishment (OR = 1.490;95%CI = 1.098–2.023; *p* = 0.011). The subjects with trajectory of resilience against anxiety presented the lowest level of emotional exhaustion and depersonalization (*p* < 0.05). The conclusion is that the pressure experienced by healthcare professionals throughout the pandemic caused different impacts on their mental health, emphasizing the dynamic nature of this condition and the need for constant monitoring and care. This finding directly affects mental health prevention and intervention measures, which remain a priority and require continuous reinforcement, especially among the most vulnerable groups.

## Introduction

The vulnerability of healthcare professionals caring for COVID-19 patients to mental health problems has been widely addressed by cross-sectional studies in many countries (1, 2). Innumerous studies report heightened levels of anxiety, depression, insomnia, post-traumatic stress, and burnout at different points in time during the COVID-19 pandemic (3, 4).

Studies conducted at the beginning of the pandemic in Brazil (5–7) report a prevalence above 36% of mental health problems among healthcare professionals, which remained high throughout the first wave (May to November 2020; (8)). Additionally, this group of professionals presented high rates of infection (61.8%) and hospitalization (10%) due to SARS-CoV-2 (9).

Thus, the need for longitudinal studies has been emphasized, given the enduring epidemiological status of COVID-19 (10). A relatively small number of longitudinal studies have been conducted, and at the same time, divergent results are reported regarding the remission or persistence of mental disorders (11).

Healthcare professionals face multiple difficulties worldwide when providing care to COVID-19 patients, including a lack of equipment, supplies, and beds (3). These difficulties have been widely reported as factors that aggravate mental health problems. Moreover, these difficulties tend to be even more pronounced in developing countries such as Brazil (12), where resources are restricted, making it challenging to meet the multiple demands of this global health crisis (13).

Valente et al. (7) note that the World Health Organization reports that, after the United States, Brazil presents the highest number of COVID-19 diagnoses in the American continent. Such a context is marked by a lack of personal protective equipment, low compliance to social distancing measures, and the low availability of diagnostic tests, especially at the beginning of the pandemic. Pereira-Lima et al. (14) report that working in highly demanding situations and limited resources (a condition faced by healthcare workers at the beginning of the pandemic) led the workforce to experience exhaustion (emotional exhaustion and depersonalization), impacting the quality of the service. De Kock et al. (15) also state that exposure to COVID-19 patients (frontline workers) and remaining in such an environment for long hours aggravates these workers’ mental health even further.

Additionally, individual and institutional factors and the macro-specific conditions of the pandemic progression (i.e., the number of new cases and deaths) influenced the health context, further compromising the workers’ mental health when the pandemic peaked or worsened (16). Because of the high incidence and mortality rates in Brazil, which reached approximately 540,000 cases and 21,000 deaths/week in the period known as the second wave (National Council of Health Secretaries - CONASS), Brazil stood out together countries such as the United Kingdom, Italy, Spain and the United States (17).

Therefore, monitoring this context is essential, especially to implement strategies to preserve human resources in the health field and minimize personal impacts (18). Hence, identifying the health workers’ mental health trajectories during the pandemic is relevant to planning preventive measures and treatments. For example, a 14-month longitudinal study conducted in Italy (11) reports that the incidence rate of mental disorders raised to 9%, while the persistence of these conditions reached 24% (post-traumatic stress).

Given the previous discussion, this study aims to monitor the progression of symptoms of mental health burden (depression, anxiety, insomnia, post-traumatic stress and burnout outcomes) among healthcare professionals caring for COVID-19 patients in Brazil during the two waves of the pandemic (May 2020 to August 2021), considering four different points in time and the number of new cases. Additionally, we intend to verify whether the different mental health trajectories (resilient, remittent, incident and persistent) are related to current burnout symptoms.

## Methods

This longitudinal study (MENTALvid) adopted a non-probabilistic sample of Brazilian frontline COVID-19 workers from different health fields. These workers were systematically monitored from the beginning of the pandemic in Brazil at different points in time. They were initially assessed every 2 weeks for a period of 90 days (the first assessment was in May 2020 - baseline), which coincided with the expansion of transmission and the peak of the virus’ first wave in Brazil (19). The last assessment of this phase occurred in August 2020 (D90), which corresponded to the end of the first wave and transition to the second wave; epidemiological data show that the number of new cases and deaths dropped at the time. Another assessment (D270) occurred in December 2020, when the new cases in Brazil indicated the peak of the second wave, given the rapid growth and predominance of the Gamma variant. Finally, a new assessment was conducted (D450 - August 2021) in a context that indicated the end of the second wave in Brazil, which was associated with the vaccination’s positive impact (19).

The participants were recruited online, through social media (Facebook, Instagram, WhatsApp), traditional media (television and radio), and by contacting class councils and relevant health institutions from different regions in Brazil. Participation was voluntary and subject to signing a free and informed consent form. The inclusion criteria in the study were: being aged 18 years or older, being a health professional (physician, nurse, nursing technician, radiology technician, psychologist, physiotherapist, nutritionist, speech therapist, dentist, pharmacist, occupational therapist and assistant social worker) active in the COVID-19 pandemic and completely fill in the data related to the baseline phase of the study. Participants who did not digitally sign the informed consent form were excluded. For the follow-up, all subjects included at the baseline phase received, *via* email and personal WhatsApp, a personalized link to access the Redcap platform and fill in the instruments corresponding to the study phase. It was submitted to and approved by the Institutional Review Board (Process 4,032,190).

### Instruments

*Generalized Anxiety Disorder-7 (GAD-7)*: 7-item self-report instrument that screens anxiety-associated symptoms. It was proposed by Spitzer et al. (20) and validated in Brazil by Moreno et al. (21); its cut-off score ≥10 presents 89% of sensitivity and 82% of specificity

*Patient Health Questionnaire-9 (PHQ-9)*: 9-item self-report instrument to assess depression symptoms. It was proposed by Kroenke et al. (22), and Osório et al. (23) validated it in Brazil. Its cut-off score is ≥10 and presents 100% sensitivity and 98% of specificity

*Posttraumatic Stress Disorder Checklist for DSM-5 (PCL-5)*: self-report instrument used to assess posttraumatic stress disorder symptoms using criteria established by the DSM-5. Its short version (8 items), translated, adapted, and psychometrically assessed by Osório et al. (24) and Pereira-Lima et al. (25), was used. Its cut-off point is ≥14 and presents sensitivity equal to 0.97 and specificity equal to 0.61 (26)

*Insomnia Severity Index (ISI)*: 7-item self-report instrument that assesses insomnia severity in the last 2 weeks. It was adapted and validated in Brazil by Castro (27), with a cut-off point ≥8 and sensitivity of 73%, and specificity of 80% to detect positive and negative cases of chronic insomnia.

*Abbreviated Maslach Burnout Inventory – Human Services Survey (aMBI-HSS)*: aims to assess burnout syndrome based on the dimensions of emotional exhaustion, depersonalization, and personal fulfillment. Its abbreviated version, proposed and validated among health professionals (28, 29), was used. Cut-off scores ≥9 indicate emotional exhaustion, ≥6 depersonalization, and ≥10 indicate professional accomplishment.

### Procedures

Data were collected and organized using the REDCap (Research Electronic Data Capture) platform. The participants received an electronic link generated by this platform’s SURVEY application to access the questionnaires. As previously mentioned, data were collected at four different points in time (baseline, D90, D279, and D450). A total of 1,522 participants accessed the platform; however, 916 completed the baseline assessment. Thus, 916 were invited to participate in the study’s remaining phases.

### Data analysis

Data were statistically analyzed using Statistical Package for the Social Sciences. The first stage included performing a descriptive analysis and tests to compare the socio-demographic information at the different phases (Chi-square and ANOVA). The percentage of subjects whose scores were above the recommended for each instrument in the baseline assessments were compared with each other evaluations by the Chi-square test. In the second stage, only symptoms of mental health burden presenting statistically significant changes during the pandemic were considered in the analysis. Hence, binary logistic regression analyses were conducted to assess associations between the incidence of new cases and these symptoms. The weekly average of new cases was calculated for the entire period of the first two waves (March 2020 to December 2021), considering figures above and below the weekly average, estimated at 216,000 new cases. The official number of new cases was reported by the COVID-19 Panel of the Strategic Information Center for SUS State Management, maintained by the National Council of Health Secretaries (30).

The third stage included assessing each participant’s outcomes (individual progression) concerning symptoms of mental health burden (i.e., anxiety, emotional exhaustion, depersonalization, professional accomplishment), comparing the baseline scores (having positive symptoms for the outcome or not) with the final assessment. The participants were assigned to one of four categories: resilient (negative symptoms at the baseline and final assessment), remittent (positive symptoms at the baseline but negative at the end), incident (negative symptoms at the baseline but positive in the final assessment), and persistent (positive symptoms in both assessments). Finally, we conducted a new binary logistic regression analysis to verify whether the four categories concerned anxiety (resilient, remittent, incident, and persistent) were associated with burnout.” The odds ratio was calculated with a 95% confidence interval. No imputation was made for missing data, and all the statistical tests were conducted considering a 0.05 significance level.

## Results

The sample was predominantly composed of women aged between 36 and 38. Approximately 40% of the sample was from the nursing field (nurses and nursing technicians), while the remaining participants were from the medical or related fields (e.g., psychologists, physiotherapists, and dentists, among others). Despite some losses, the sample’s profile did not change over the assessments, enabling data comparison (see Table 1). Most participants were frontline workers from public tertiary hospitals, considered reference centers for treating patients with COVID-19 in Brazil.

**Table 1:** Participants’ socio-demographic characteristics at the four points in time.

Variables	Follow-up				Statistics
	Baseline	90	270	450	
***N* (% Dropout rate)**	916 (0%)	209 (77.2%)	145 (84.2%)	177 (80.7%)	
**Gender *N* (%)**					
Female	730 (79.7)	166 (79.4)	198 (80.8)	144 (81.3)	Chi-square = 0.374
Male	186 (20.3)	43 (20.6)	47 (19.2)	33 (18.7)	*p* = 0.541
**Age**					
Mean	36.1	38.3	37.7	37.7	ANOVA *F* = 0.701
(SD)	(31.5)	(09.5)	(09.7)	(09.3)	*p* = 0.551
**Occupation *N* (%)**					
Nurse	376 (41.0)	76 (36.4)	85 (34.7)	63 (35.6)	Chi-square = 2.698
**Other**	540 (59.0)	133 (63.6)	160 (65.3)	114 (64.4)	*p* = 0.100

**Figure 1:**
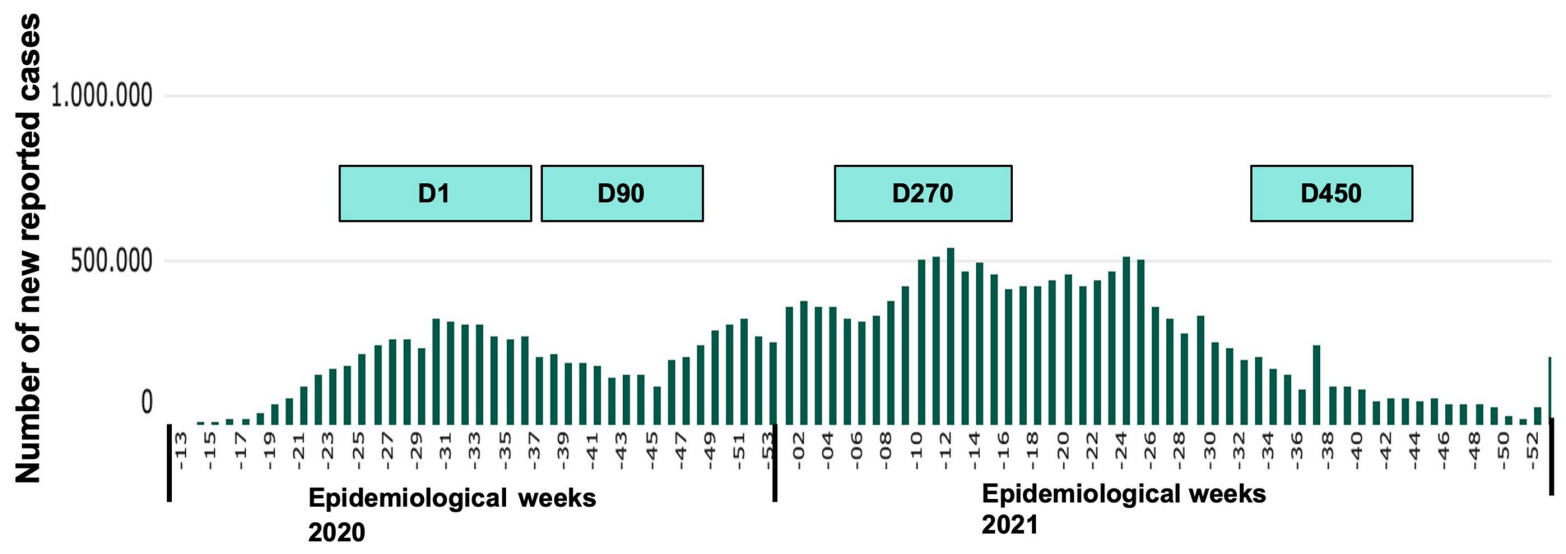
Data collection at different points in time, considering the number of new COVID-19 cases (adapted from CONASS - National Council of Health Secretaries https://www.conass.org.br/painelconasscovid19/).

Figure 1 present the incidence of new cases during recruitment (30). The comparisons of the self-reported scales in the four assessments showed significant differences between them in anxiety (*p* = 0.003), emotional exhaustion (*p* = 0.009), depersonalization (0.0450), and professional accomplishment (*p* < 0.001). No significant changes (*p* > 0.05) were found for the depression, insomnia, or post-traumatic stress outcomes. These data and the percentage of subjects whose scores were above the recommended for each instrument were showed in Table 2. A decrease in anxiety levels was found in D90. This period coincides with a decline in new cases (end of the first wave), though higher rates were found in the remaining phases. The symptoms for emotional exhaustion concerning burnout increased in D270, the period with the highest incidence of cases, and then returned to the baseline levels in the D450 assessment. Depersonalization levels increased in this phase, with a further decrease in new cases (end of the second wave). Professional accomplishment symptoms decreased from D90 onwards and did not return to baseline.

**Table 2:** Percentage of subjects whose scores were above the cut-off for each instrument in the different assessments.

Measures	Follow-up			
	Baseline	90	270	450
**Anxiety**				
Number of respondents	916	209	245	177
% of GAD 7 ≥ 10	43.3	30.6*	42.9	35.6
**Depression**				
Number of respondents	916	205	242	175
% of PQH 9 > 10	40.2	35.1	45.0	40.0
**Insomnia**				
Number of respondents	916	201	237	171
% of ISI ≥ 8	61.5	59.2	60.8	55.0
**Posttraumatic stress disorder**				
Number of respondents	916	201	237	172
% of PCL 5 > 13	36.0	35.3	35.4	35.5
**Burnout (emotional exhaustion)**				
Number of respondents	916	202	240	174
% of AMBI-EE > 8	36.7	38.1	47.9*	43.7
**Burnout (depersonalization)**				
Number of respondents	916	202	240	174
% of AMBI-D > 5	18.2	22.3	21.7	27.0*
**Burnout (professional accomplishment)**				
Number of respondents	916	202	240	174
% of AMBI-PA > 9	83.0	67.3*	73.3*	71.8*

* The differences between baseline and follow-ups were statistically significant (Chi-square – *p* < 0.05).

The average incidence of new cases in the period addressed in this study was estimated at 216,000/week. The symptoms were above average at the baseline and in D270 but below average in the D90 and D450 periods. Thus, the association between the incidence of new cases (above or below the average) with anxiety and burnout symptoms revealed that a higher number of new cases was associated with a greater frequency of anxiety (odds ratio = 1.467; 95%CI = 1.109–1.941; *p* = 0.007) and professional accomplishment (odds ratio = 1.490; 95%CI = 1.098–2.023; *p* = 0.011) among healthcare professionals. However, a higher number of new cases did not predict high symptoms of Emotional exhaustion (odds ratio = 0.964; 95%CI = 0.732–1.269; *p* = 0.794) or Depersonalization (odds ratio = 0.936 95%CI = 0.691–1.268; *p* = 0.669).

Table 3 presents the participants’ outcomes distributed into four groups, considering the baseline and D450 assessments. The resilient group, i.e., individuals with negative symptoms for anxiety, depersonalization, and emotional exhaustion at the baseline and final assessment, was the most predominant. The incidence and persistence rates for anxiety and emotional exhaustion also draw attention, as well as the percentage of subjects whose trajectory indicates loss of professional accomplishment (about 30%).

**Table 3:** Clinical characteristics of the four groups regarding anxiety, depersonalization, emotional exhaustion, and professional accomplishment.

Diagnosis at the		*N* (%) of participants with			
Baseline	Endpoint	Anxiety	Depersonalization	Emotional exhaustion	Non-professional accomplishment
No	No	82 (46.3)^(A)^	115 (66.14)^(A)^	77 (44.3)^(A)^	112 (64.4)^(A)^
Yes	No	32 (18.1)^(B)^	12 (6.9)^(B)^	21 (12.1)^(B)^	32 (18.4)^(B)^
No	Yes	26 (14.7)^(C)^	27 (15.5)^(C)^	26 (14.9)^(C)^	13 (7.5)^(C)^
Yes	Yes	37 (20.9)^(D)^	20 (11.5)^(D)^	50 (28.7)^(D)^	17 (9.8)^(D)^

(A) Resilient; (B) Remittent; (C) Incident; (D) Persistent.

**Figure 2:**
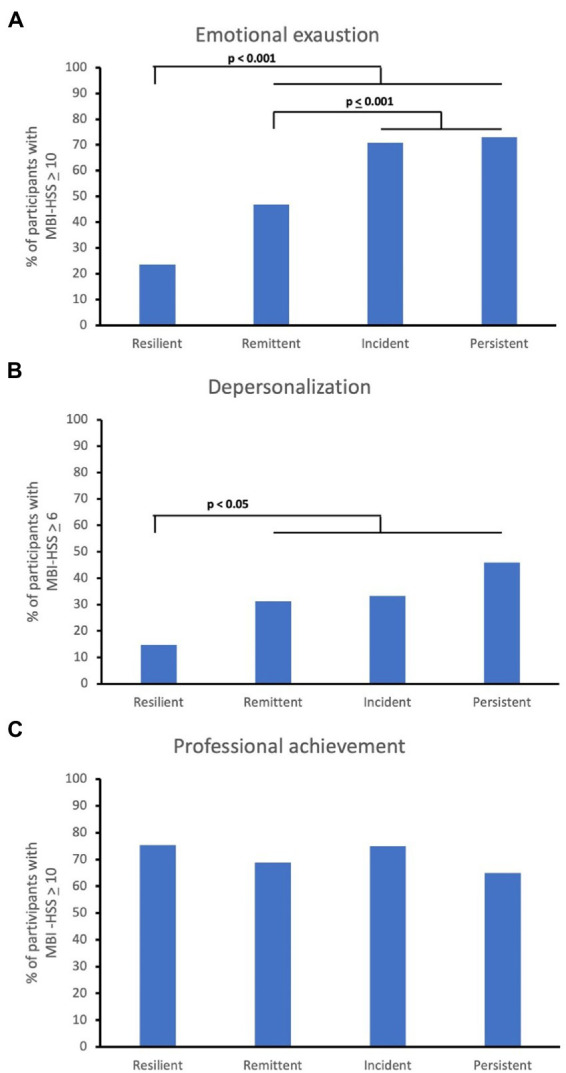
Burnout symptoms considering the distribution of individuals according to the anxiety outcome (baseline vs. D450); **(A)** Emotional exhaustion; **(B)** Depersonalization; **(C)** Professional achievement.

Additional analyzes were performed to assess the impact of anxiety on burnout symptoms. Thus, the percentage of individuals with burnout symptoms was calculated for each group, considering the individuals’ distribution according to the anxiety outcome (Figure 2; Supplementary material 1).

The resilient group against anxiety presented the lowest levels of emotional exhaustion and depersonalization (*p* < 0.05). Additionally, the regression analyses showed that not belonging to the anxiety-resilient group increased the likelihood of having emotional exhaustion at the end of the second wave by 5.8 times (D450; odds ratio = 5.800; 95%CI = 2.965–11.347; *p* < 0.001). On the other hand, belonging to the anxiety-resilient group is associated with a tendency toward lower levels of depersonalization (D450: odds ratio = 2.425; 95%CI = 0.919–6.400; *p* = 0.073). Additionally, it is not associated with emotional fulfillment at this stage (D450: odds ratio = 0.724; 95%CI = 0.371–1.413; *p* = 0.343).

## Discussion

This study presented the mental health trajectories of health professionals throughout the two waves of the COVID-19 pandemic in Brazil (from May 2020 to August 2021, with two peaks and consequent declines in the number of new cases and deaths). This study differs from most longitudinal studies because such studies are restricted to assessing the impact and progression of mental distress in the early stages of the pandemic. Furthermore, the COVID-19 pandemic is peculiar and unprecedented, given its duration, extension, and potential for recurrent trauma, with unknown consequences for healthcare professionals’ mental and physical health in the medium-term (31, 32). Therefore, long-term assessments, such as the one presented here, addressing different points in time are essential to identify the most appropriate response to the demands of this group of workers.

According to Magnavita et al. (4), the situations and pressure experienced by health professionals differed throughout the pandemic, in which emerging problems coupled with the stressors usually experienced in health practice. The authors mentioned above highlight the challenge of devising strategies to treat a novel, unknown disease, expanding occupational safety procedures, minimizing transmission, and dealing with ethical dilemmas associated with the scarcity of resources, which prevented the delivery of appropriate patient care in the first wave of the pandemic.

As the pandemic progressed, the situation changed with the emergence of other stressors such as work overload, the impacts of social isolation, and compassion fatigue, among others. However, positive changes emerged with the vaccine being made available. It granted greater protection for those exposed to typical working conditions and enabled better control of the pandemic and the unfavorable outcomes resulting from the disease. Note that at the last data collection (D450), 50% of the Brazilian population had taken at least one dose of the vaccine, and 22% had already completed the vaccination schedule (33).

The high prevalence (>36%) of mental distress among Brazilian health professionals at the beginning of the first wave of the pandemic was highlighted in a previous study (6), and remained high at its end (8). In this study, we expanded the follow-up, including two new points in time, which coincided with the peak of new cases of the second wave and the end of it. As the pandemic progressed (number of new cases and deaths), an association between anxiety and burnout symptoms was found, highlighting the dynamic nature of this condition and a need for constant monitoring and care.

In particular, there was a significant decrease in anxiety levels at the end of the first wave, which is consistent with the results of some studies conducted worldwide (34–36). According to the literature (32, 34), such a decrease may be attributed to greater control over the pandemic, more knowledge and resources for treating patients, greater familiarity with the new working routine, and decreased stigma associated with healthcare workers. Our findings reinforce the association between anxiety and the number of new cases and deaths caused by COVID-19.

However, the second wave caused anxiety to return to previous levels. Heightened anxiety possibly reflected the potential effects of work overload, the depletion of personal resources, occupational stress, and increased alertness to the possibility of new waves given the unpredictable course of the pandemic (32, 37–39). Higher symptoms of emotional exhaustion corroborate these findings related to a higher number of new cases and deaths. However, these symptoms decreased when the situation became more manageable, reflecting the dynamic nature of these conditions and reiterating the need for constant monitoring and care.

Dufour et al. (1) also note that the psychological distress the professionals experienced when the pandemic was under greater control might be explained by fatigue, absence of vacations, and accumulated stress. Additionally, we should remember the oscillations in anxiety and emotional exhaustion symptoms, which were accompanied by a loss of professional accomplishment. The latter decreased at the end of the first wave and did not return to the baseline levels.

Furthermore, as the epidemic became chronic, an increase in depersonalization levels was found for the first time. As a result, feelings of insensitivity and negativism towards patients emerged, potentially harming the quality of care delivery. Lack of motivation at work has already been found in previous pandemics (40, 41). However, Leeuwen et al. (42) note that an increase in the social appreciation of health professionals in the current context may have promoted greater motivation, especially in the early stages of the fight against the virus.

Additionally, some studies report other unfavorable outcomes associated with the occupational context as the pandemic progressed. For example, Magnavita et al. (4) note that 1 year after the COVID-19 pandemic began, 22% of the workers participating in their study were dissatisfied with their jobs, and 41% wished to resign. Luceño-Moreno et al. (43) performed an assessment 6 months after the pandemic onset, highlighting an increase in burnout symptoms. Wynter et al. (44) report an increase in overtime and conflicts at work.

The workers’ trajectories revealed that the anxiety-resilient group was more numerous in all the indicators. This finding is quite positive and aligns with the results reported by studies assessing trauma-related outcomes (45). It is also in line with the results reported by Dufour et al. (1, 31), Rossi et al. (11), and Peccoralo et al. (46), who assessed the trajectories of health professionals working in the COVID-19 pandemic. However, it is essential to note the persistence and incidence of different mental health conditions, especially anxiety and burnout. The indicators emerge as a warning, pointing out the non-transient effects of the pandemic. Indeed, high levels of depression, insomnia, and post-traumatic stress have remained constant in this context of risk.

Finally, the association between anxiety and burnout stands out, considering that non-resilient healthcare professionals experienced greater emotional exhaustion and depersonalization at the end of the second wave. These findings emphasize the importance of psychological support and other health actions to minimize anxiety symptoms and promote decreased burnout, considering the negative repercussions of these outcomes for the health staff and patients (47).

This study’s results align with those reported in other countries (e.g., 32, 48, 49). Hence, the pandemic did not have a one-off impact on the context of Brazilian workers; instead, its ongoing nature is emphasized. This finding is relevant because it directly impacts mental health prevention measures and interventions. Such strategies remain a priority and require continuous reinforcement, especially for groups with more vulnerable trajectories (42). According to Dufour et al. (1, 31), these measures can make a difference in the progression of more favorable trajectories, preventing, first and foremost, chronic diseases. We believe such care is essential for maintaining a healthy and employable workforce, which is crucial for a sustainable health system. Thus, the implementation and maintenance of care procedures remain a priority.

Given this context, continually assessing healthcare workers’ mental health seems crucial. Kromydas et al. (50) note that healthcare workers are one of the professions most intensively impacted by the pandemic. The reason is that the prospects of the pandemic are still uncertain, and we need to deepen our knowledge of the predictors of different trajectories (1, 31).

Finally, given the pandemic’s unstable nature and the workers’ emotional condition, self-monitoring is essential to provide timely acute care and support measures to deal with psychological distress. Additionally, institutions should implement measures to preserve the quality and effectiveness of the service health professionals provide, especially in middle-income countries like Brazil (47).

The conclusion is that the pressure experienced by healthcare professionals throughout the pandemic caused different impacts on their mental health, emphasizing the dynamic nature of this condition and the need for constant monitoring and care. This finding directly affects mental health prevention and intervention measures, which remain a priority and require continuous reinforcement, especially among the most vulnerable groups.

This study’s limitations include the use of self-report measures, as such measures depend on the participants’ ability to assess and report their conditions and symptoms accurately; bias related to convenience sampling; online data collection, using screening instruments without diagnostic measures; and sample losses that imply restricted representativeness, involving factors such as the management of and access to electronic devices, and occupational conditions related to quitting or the desire to quit working in the healthcare field. In addition, it was decided to evaluate only the presence of some symptoms related to symptoms of mental health burden, that is, those most commonly considered in previous studies with similar objectives. However, other symptoms can and should be considered, which can also portray the impacts of the pandemic on the mental health and quality of life of these professionals.

## Data availability statement

The raw data supporting the conclusions of this article will be made available by the authors, without undue reservation.

## Ethics statement

The studies involving human participants were reviewed and approved by Comitê de Ética em Pesquisa do Hospital das Clínicas da Faculdade de Medicina de Ribeirão Preto - USP - Process 4.032.190. The patients/participants provided their written informed consent to participate in this study.

## Author contributions

FO, AZ, JC, JH, KP-L, and SL: conception and design and substantial contributions to drafting the article or revising it critically for important intellectual content. FO, IS, AZ, and SL: collect, analysis, and interpretation of data. FO, AZ, IS, JC, JH, KP-L, and SL: final approval of the version to be published. All authors contributed to the article and approved the submitted version.

## Funding

This work was supported by Ministry of Science, Technology, Innovation and Communications; Ministry of Health of Brazil – MoH; and National Council for Scientific and Technological Development – CNPq (Process number 401058/2020-4).

## Conflict of interest

The authors declare that the research was conducted in the absence of any commercial or financial relationships that could be construed as a potential conflict of interest.

## Publisher’s note

All claims expressed in this article are solely those of the authors and do not necessarily represent those of their affiliated organizations, or those of the publisher, the editors and the reviewers. Any product that may be evaluated in this article, or claim that may be made by its manufacturer, is not guaranteed or endorsed by the publisher.
