# Supplementary material for: Mental health trajectories of Brazilian health workers during two waves of the COVID-19 pandemic (2020–2021)
Source: Front Psychiatry. 2023 Mar 23;14:1026429. doi: 10.3389/fpsyt.2023.1026429 (PMC10076806; doi:10.3389/fpsyt.2023.1026429)
Supplement: Supplementary file 1 [file Table_1.docx]

SM1 – Burnout indicators considering the distribution of individuals according to the anxiety outcome (baseline *vs.* D450)- Figure 2

|  | **% of participants with the three dimentions**  **of  burnout > cut-off scores** | | |
| --- | --- | --- | --- |
| **Anxiety categories** | **Dimentios of burnout** | | |
|  | **Emotional**  **exaustion** | **Professional achievement** | **Depersonalization** |
| **Resilient** | 23.5 | 75.3 | 14.8 |
| **Remittent** | 46.9 | 68.8 | 31.3 |
| **Incident** | 70.8 | 75.0 | 33.3 |
| **Persistent** | 73.0 | 64.9 | 45.9 |
